# RNA extraction method is crucial for human papillomavirus E6/E7 oncogenes detection

**DOI:** 10.1186/s12985-017-0720-x

**Published:** 2017-03-09

**Authors:** Nerea Fontecha, Maria Carmen Nieto, Daniel Andía, Ramón Cisterna, Miren Basaras

**Affiliations:** 1 Department of Immunology, Microbiology and Parasitology, Medicine and Odontology Faculty, University of Basque Country (UPV/EHU), Sarriena auzoa, 48940 Leioa-Bizkaia, Spain; 2 Clinical Microbiology and Infection Control Department, Basurto University Hospital, Bilbao, 48013 Spain; 3 Department of Obstetrics and Gynaecology, Basurto University Hospital, Bilbao, 48013 Spain

**Keywords:** High-risk HPV, E6/E7 mRNA, Oncogenes, RNA extraction methods, Cervical cancer biomarker

## Abstract

**Background:**

Human papillomavirus (HPV) DNA testing plays a main role in the management of cervical cancer, however to improve the specificity in cervical screening, there is a need to develop and validate different approaches that can identify women at risk for progressive disease.

Nowadays, mRNA expression of viral E6 and E7 HPV oncogenes stands up as a potential biomarker to improve cervical screening. We aimed to validate a method for RNA extraction, detect HPV mRNA expression and, assess the relationship between E6/E7 mRNA expression and pathology of patients’ lesions and progression.

**Methods:**

This study included 50 specimens that had been previously genotyped as HPV16, 18, 31, 33 and/or 45. Cervical swabs were extracted with three different RNA extraction methods -Nuclisens manual extraction kit (bioMérieux), High Pure Viral RNA Kit (Roche) and RNeasy Plus Mini kit (Qiagen)-, and mRNA was detected with NucliSens EasyQ HPV version 1 test (bioMérieux) afterwards. Association of oncogene expression with pathology and lesion progression was analyzed for each extraction method.

**Results:**

E6/E7 mRNA positivity rate was higher in samples analyzed with bioMérieux (62%), followed by Roche (24%) and Qiagen (6%). Women with lesions and lesion progression showed a higher prevalence of viral RNA expression than women that had not lesions or with lesion persistence. While bioMérieux revealed a higher sensitivity (77.27%), Roche presented a higher PPV (75%) and an increased specificity (89.28%).

**Conclusions:**

Extraction methods based on magnetic beads provided better RNA yield than those based in columns. Both Nuclisens manual extraction kit (bioMérieux) and High Pure Viral RNA Kit (Roche) seemed to be adequate for E6/E7 mRNA detection. However, none of them revealed both high sensitivity and specificity values. Further studies are needed to obtain and validate a standard gold method for RNA expression detection, to be included as part of the routine cervical screening program.

## Background

High-risk Human papillomavirus (HR-HPV) infection persistence is a necessary feature to develop cervical cancer, which is the third most common cancer in women worldwide, and the second most common cancer in developing regions [1–3]. Cancer incidence and mortality have decreased significantly since the introduction of cancer screening, which started with the Papanicolau test in 1940’s.

HPV genome has two oncogenes, E6 and E7, whose expression increased when these genes are deregulated [4]. Oncogene deregulation affects the normal running of the host cell increasing cell cycle entry and loss of differentiation over the cervical epithelium [5, 6]. The integration of HPV DNA into the host genome is an important event in cancer development and in malignant transformation of cervical lesions [7]. In patients with squamous cell carcinomas, the virus genome usually is integrated into host cell and when HPV circular genome integrates into host cell genome, E2 gene ORF (Open Reading Frame) is broken to linearize virus genome so E2 function is altered [8]. In fact, HPV E2 gene regulates HPV late gene expression [9]. Namely, HPV viral oncoprotein expression differs among infection state (active, latent, or persistent) [9–11].

Nowadays, cervical cancer screening is based on cytology and/or HPV DNA detection [12, 13]. HPV DNA detection reveals a high prevalence of transient and asymptomatic infections but does not give any information about the infection state [9]. Therefore, new approaches (biomarkers) are being studied to improve cervical screening, minimize patient anxiety, over-referral to colposcopy and treatment, as well as to decreased related costs.

One of the most promising cervical cancer biomarkers is the mRNA expression of viral E6 and E7 oncogenes, since its association with the severity of cervical lesions is well described in the literature [14]. In 2007, bioMérieux launched NucliSens EasyQ HPV version 1 test, a real-time nucleic acid amplification and multiple detection assay, that qualitatively detects the expression of the oncogenic E6/E7 mRNA from the five most common high HR-HPV genotypes: HPV16, 18, 31, 33 and 45 [15, 16].

The present study aimed to assess E6/E7 mRNA as a possible biomarker for cervical cancer by analyzing the relationship between HPV E6/E7 mRNA expression and the pathology and evolution of different lesions. As some authors have reported the importance of RNA input (both quality and quantity) prior to any HPV RNA assay [17–19], we aimed to compare three different RNA extraction methods in order to evaluate the impact of extraction in RNA expression detection.

## Methods

### Patients and clinical samples

From 2010 to 2014, all women (*n* = 912) attending Consultation of Sexually Transmitted Diseases and Gynaecological consultation at Basurto University Hospital (Basque Country, Spain) were remitted to Clinical Microbiology and Infection Control Department to analyze their samples, when a possible HPV infection was suspected.

Samples were collected following endo/ectocervical swabbing with a cytobrush and, stored in PreservCyt (Hologic. Inc., Marlborough, MA) transport medium at room temperature until HPV presence or absence was studied.

Lesions were classified by pathologists following Bethesda system: 1) Negative (no lesion was found) 2) Low-grade squamous intraepithelial lesion (LSIL) - atypical squamous cells of undetermined significance (ASCUS) included- and, 3) High-grade squamous intraepithelial lesion (HSIL).

Cytology was repeated 2 years after samples collection and, results were categorized into three groups depending on lesion progression: 1) Persistence: women whose cytological results had not changed in the last 2 years, 2) Progression: women whose cytological results showed a worsened process, and 3) Regression: women who had cleared signs of infection.

Written and informed consent was obtained from participants.

The study adhered to the declaration of Helsinki and was approved by Ethical Committee of Clinical Research of Euskadi (Code: PI2014016).

All methods were carried out in accordance with the approval guidelines.

### Genotype detection

HPV genotyping was performed using Cobas® HPV Test (Roche Molecular Diagnostics, Mannheim, Germany) and Beta-globin was used as an internal control to ensure specimen adequacy.

Cobas® HPV Test detects up to 14 HR-HPV genotypes, identifying HPV16 and 18 specifically, while detecting “other HR-HPV genotypes” (31, 33, 35, 39, 45, 51, 52, 56, 58, 59, 68, 66) concurrently.

Samples that had been positive for “other HR-HPV genotypes” with Cobas® HPV Test were therefore, subjected to Linear Array HPV Genotyping Test kit (Roche Molecular Diagnostics, Mannheim, Germany), to elucidate which specific HR-HPV genotypes were present in each specimen. Linear Array HPV Genotyping Test is a line-blot assay that detects 37 HPV genotypes (6, 11, 16, 18, 26, 31, 33, 35, 39, 40, 42, 45, 51, 52, 53, 54, 55, 56, 58, 59, 61, 62, 64, 66, 67, 68, 69, 70, 71, 72, 73, 81, 82, 83, 84, IS39, and CP6108).

All samples from women who were positive for HPV16, 18, 31, 33 and/or 45 were included for further RNA analysis (*n* = 50).

### RNA extraction

RNA was extracted with three different kits according to manufacturer’s instructions: 1) Nuclisens manual extraction kit (bioMérieux, Marcy l’Etoile, France)-RNA was extracted from 200 μl of samples by NucliSENS Lysis Buffer and NucliSENS® miniMAG® and eluted in 60 μl of elution buffer-, 2) High Pure Viral RNA Kit (Roche Molecular Diagnostics, Mannheim, Germany) - RNA was extracted from 200 μl of sample and eluted in 50 μl of Elution Buffer- and, 3)

RNeasy® Plus Mini kit (Qiagen, Hilden, Germany) -RNA was extracted from 400 μl of sample and eluted in 50 μl of RNase-Free Water-.

RNAse-Free water was used as negative control in each RNA extraction. Eluted RNA was stored at -20 °C until E6/E7 analysis (never exceeding 2 weeks) and at -80 °C, afterwards.

### E6/E7 oncogene expression

Oncogene expression was analyzed in each sample three times (one for each RNA extraction method). Those samples that had been genotyped as positive for several targeted HPV genotypes (HPV16, 18, 31, 33 and/or 45) were analyzed once for each genotype and extraction method.

E6/E7 mRNA was detected by NucliSens EasyQ HPV test version 1 (bioMérieux, Marcy l’Etoile, France) following manufacturer’s instructions, including an internal control, human U1 small nuclear ribonucleoprotein specific protein A (U1A), to assess mRNA expression quality. Samples with no RNA expression were re-extracted and oncogene expression was analyzed once again.

### Data analysis

Positive predictive value (PPV), Negative predictive value (NPV), sensitivity and specificity were calculated for each extraction method. The concordance between different extraction methods was studied by the relative observed agreement (Pr(*a*)) and Cohen’s kappa coefficient (κ). Statistical results were categorized according to Landis and Koch classification: values lower than 0 indicates no agreement, from 0 to 0.20 slight agreement, from 0.21 to 0.40 fair agreement, from 0.41 to 0.60 moderate, from 0.61 to 0.80 substantial, and from 0.81 to 1 as nearly perfect agreement [20].

Association of oncogene expression with pathology and lesion progression was also analyzed for each extraction method.

## Results

### Patients’ data

The age range of enrolled women (*n* = 50) varied from 19 to 63 years (mean age34.96 ± 10.63 years). Among these women, 42% (21/50) were infected with only one HPV genotype (single HPV infection) while 58% (29/50) were infected with more than one HPV genotypes (multiple HPV infection). However, only 4/29 multiple infections revealed the presence of more than one HPV targeted genotype (HPV16, 18, 31, 33 and/or 45). All four samples were multiple infections of 2 HPV genotypes.

The most detected genotype was HPV16 (64%, 32/50) followed by HPV45 (14%, 7/50), HPV18 (12%, 6/50), HPV31 (10%, 5/50) and HPV33 (6%, 3/50).

According to Bethesda system, 8% of samples were classified as HSIL (4/50), 36% (18/50) as ASCUS or LSIL and 56% (28/50) as negative.

### Nuclisens manual extraction kit (bioMérieux)

E6/E7 mRNA positivity rate was 62% (31/50). Although the most detected genotype after DNA analysis was HPV16, the most expressed genotype was HPV33 followed by HPV18, HPV16, HPV31 and HPV45 (Table 1).

**Table 1:** E6/E7 mRNA expression of each genotype and oncogenes positivity rate according to three RNA extraction methods

Genotype (n° of samples)	E6/E7mRNA expression (n°, %)		
	Nuclisens manual extraction kit (bioMérieux)	High Pure Viral RNA Kit (Roche)	RNeasy Plus Mini kit (Qiagen)
HPV 16 (32)	22/68.75	6/18.75	2/6.25
HPV 18 (6)	5/83.33	3/50.00	0/0.00
HPV 31 (5)	2/40.00	1/20.00	1/20.00
HPV 33 (3)	3/100.00	2/66.67	0/0.00
HPV 45 (7)	2/28.57	2/28.57	0/0.00
E6/E7mRNA positivity rate (%)	62	24	6

The results obtained by mRNA analysis showed concordance with HPV-types from the genotyping methods for all samples but one. This sample was classified as positive for HPV16 and 45 genotypes according to DNA genotyping while oncogene expression revealed positivity for HPVs 16, 45 and 31.

**Fig. 1:**
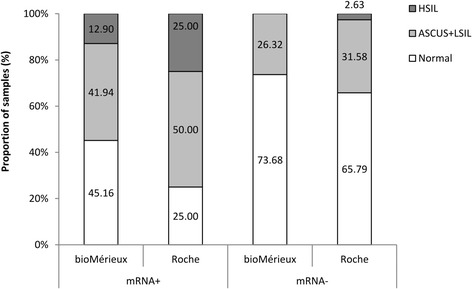
Pathology according to E6/E7mRNA expression in two different extraction methods. E6/E7 mRNA rates: positivity rate for E6/E7 mRNA expression (mRNA+) and negativity rate for E6/E7 mRNA expression (mRNA-). Pathology was classified into three groups: 1) Normal (no lesion), 2) Atypical squamous cells of undetermined significance (ASCUS) or low-grade squamous intraepithelial lesion (LSIL) and, 3) High-grade squamous intraepithelial lesion (HSIL). bioMérieux extraction method was Nuclisens manual extraction kit; Roche extraction method was High Pure Viral RNA Kit

Among E6/E7 mRNA positive samples (31/50), 54.84% (17/31) belonged to women who presented lesions (41.94% to women with ASCUS or LSIL and 12.90% to women with HSIL), while for mRNA negative specimens (19/50), 73.68% (14/19) corresponded to women without cervical lesions. All women with high-grade lesions were classified as positive for oncogene expression (Fig. 1).

**Fig. 2:**
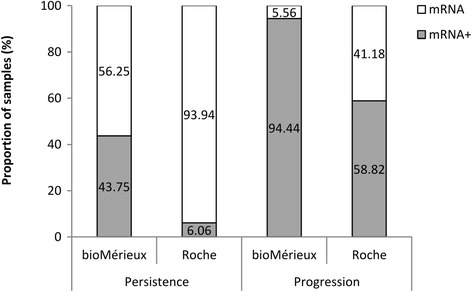
Lesion progression according to E6/E7 mRNA expression in two different extraction methods. E6/E7 mRNA rates: positivity rate for E6/E7 mRNA expression (mRNA+) and negativity rate for E6/E7 mRNA expression (mRNA-). Lesion evolution was categorized into two groups: 1) Persistence: women whose cytological results had not changed in the last 2 years and, 2) Progression: women whose cytological results showed a worsened process. bioMérieux extraction method was Nuclisens manual extraction kit; Roche extraction method was High Pure Viral RNA Kit

Analysis of lesion evolution revealed that 94.44% of women that had suffered from lesion progression, were positive for E6/E7 mRNA detection while the percentage dropped to 43.75% for women with lesion persistence (Fig. 2). No women showed infection regression.

### High pure viral RNA Kit (Roche)

Oncogene expression was detected in 24% (12/50) of women. The most expressed genotype was HPV33 followed by HPV18, HPV45, HPV31 and HPV16 (Table 1). The same genotypes that had been detected by HPV DNA genotyping, were detected by mRNA expression in each sample, but for one specimen (which had been genotyped as HPV16 and 45, but mRNA expression revealed presence of HPV16, 45, 31 and 33).

Nine out of 12 (75%) women who were positive for E6/E7 mRNA expression had some type of lesion (50% women with ASCUS or LSIL and 25% with HSIL) (Fig. 1).

Among samples that were negative for oncogene expression (38/50), 65.79% (25/38) belonged to women without lesion, 31.58% (12/38) to women with ASCUS or LSIL and, 2.63% (1/38) to women with high-grade lesion. Concerning lesion evolution, none of tested women had infection clearance. Only 6.06% of women with lesion persistence were positive for oncogene expression while the percentage increased up to 58.82% for women with lesion progression (Fig. 2).

### RNeasy® Plus Mini kit (Qiagen)

Only 6% of all specimens (3/50) analyzed after Qiagen extraction, showed mRNA expression. No significant relationship (*p* > 0.05) was detected between oncogene expression and type of lesion. Due to the low number of E6/E7 mRNA positive samples, data obtained with this method was excluded for comparison with other methods.

### Comparison between bioMérieux and Roche RNA extraction methods

While the NPV was similar for both extraction methods (65.79% Roche vs. 73.68% bioMérieux), the PPV was higher for Roche than for bioMérieux (75% vs. 54.84%, respectively). Roche extraction method also showed a higher specificity (89.28% vs.50%), however, its sensitivity was lower (40.90% vs. 77.27%) when comparing to the bioMérieux extraction method.

Considering that viral oncogenes should be expressed in women with lesions but not in women without lesions, bioMérieux extraction method showed substantial and nearly perfect observed agreement whenever women presented some type of lesion (0.72 in LSILs and 1 in HSILs) whereas Roche extraction method, showed nearly perfect agreement (0.89) for women without lesion (Table 2).

**Table 2:** The relative observed agreement concerning pathology and lesion progression in two RNA extraction methods

Analysis of each extraction method separately			
	Method		The relative observed agreement Pr(*a*)
Pathology	Nuclisens manual extraction kit (bioMérieux)	No lesion	0.5
		ASCUS + LSIL	0.72
		HSIL	1
	High Pure Viral RNA Kit (Roche)	No lesion	0.89
		ASCUS + LSIL	0.33
		HSIL	0.75
Lesion progression	Nuclisens manual extraction kit (bioMérieux)	Same lesion	0.52
		Worsened lesion	0.74
	High Pure Viral RNA Kit (Roche)	Same lesion	0.95
		Worsened lesion	0.40
Comparison between biomerieux and Roche extraction methods			
E6/E7 mRNA detection rate			0.58
Pathology		No lesion	0.61
		ASCUS + LSIL	0.50
		HSIL	0.75
Lesion progression		Same lesion	0.57
		Worsened lesion	0.60

Pathology was categorized into three groups: 1) Normal (no lesion), 2) Atypical squamous cells of undetermined significance (ASCUS) or low-grade squamous intraepithelial lesion (LSIL) and, 3) High-grade squamous intraepithelial lesion (HSIL). Lesion progression was divided into two groups: 1) women whose cytological results had not changed in the last two years and, 2) women whose cytological results showed a worsened process

Analyzing lesion development, Roche extraction methods revealed a nearly perfect agreement for women that showed persistence in their lesions (0.95) whereas bioMérieux extraction method showed substantial concordance in women with worsened lesions (0.74).

When comparing the 2 RNA extraction methods against each other, a moderate relative observed agreement was seen regarding E6/E7 mRNA detection rate (0.58) and lesion development (0.57 for women that showed persistance and 0.60 for women with worsened lesions). Concerning pathology, a substantial agreement was found in women without lesion or with high-grade lesions (0.61 and 0.75, respectively) while the concordance was just moderate when studying in women with low-grade lesions (0.50).

Considering Cohen’s kappa coefficient, agreement between methods was fair regarding E6/E7 mRNA detection rate (0.26), and pathology (0.26 bioMérieux and 0.32 Roche). Concerning lesion progression, Roche extraction method showed moderate agreement (0.41) whereas bioMérieux agreement was fair (0.26) (Table 3).

**Table 3:** Cohen’s kappa coefficient concerning E6/E7 mRNA detection rate, pathology and lesion progression in two RNA extraction methods

Analysis of each extraction method separately		
	Method	Cohen’s kappa coefficient κ
Pathology	Nuclisens manual extraction kit (bioMérieux)	0.26
	High Pure Viral RNA Kit (Roche)	0.32
Lesion progression	Nuclisens manual extraction kit (bioMérieux)	0.26
	High Pure Viral RNA Kit (Roche)	0.41
Comparison between biomerieux and Roche extraction methods		
E6/E7 mRNA detection rate		0.26

## Discussion

mRNA detection has been suggested as a promising cervical cancer prognosis biomarker, as it might elucidate the state of infection in patients [21]. It is highly important to analyze all possible oncogenic types, in order to establish a successful cervical screening. We analyzed 50 patients with oncogenic HPV genotypes (HPV16, 18, 31, 33 and/or 45), and up to 62% revealed mRNA expression.

While HPV16 was the most common genotype detected in these samples, mRNA analysis revealed that other genotypes such as HPV33 and 18 were more frequently expressed. Differences in expression detection might have to do with the sensitivity and specificity of the method used. It is highly likely that very low levels of HPV transcripts might not be detected and still, those “low levels” might be enough for HPV to perform its action in the infected cells. Integration, an important event in the progression of the disease, might not be solely responsible for the progression of the disease, as presence of only episomal forms has been detected in patients with advanced cervical squamous cell carcinomas [22, 23]. However, integrated forms with overexpression of E6 and E7 leading to transformation of cells might be detected more frequently than episomal forms.

Validating a protocol for mRNA detection is essential, as false positive and/or false negatives might translate into increasing patient anxiety, over-referral to colposcopy and treatment, as well as to increasing related costs. Our aim was to compare three different RNA extraction methods (Nuclisens manual extraction kit from bioMérieux, High Pure viral nucleic acid kit from Roche and RNeasy Plus Mini kit from Qiagen) and assess the difference found when analyzing E6 and E7 oncogene expression.

Among the extraction methods, E6/E7 mRNA positivity rate was higher in samples analyzed with bioMérieux (62%, 31/50), followed by Roche (24%, 12/50) and Qiagen (6%, 3/50). These results are in agreement with other studies that compares these extraction methods for human immunodeficiency virus [24]. bioMérieux extraction method is based on cell lysis and magnetized silica dioxide particles while Roche and Qiagen extraction methods are based on column techniques. It is well described in the literature that extraction methods based on magnetic beads provide a higher RNA yield and purity, and less inhibitors compared to spin columns [25, 26]. Due to the low rate obtained with Qiagen extraction, this method was excluded for further analysis.

Both bioMérieux and Roche extraction methods showed similar results when comparing RNA expression, type of lesion and evolution. Women with lesions showed a higher prevalence of viral RNA expression (54.84% and 75%, for bioMérieux and Roche, respectively) while women that had not lesions revealed a lower prevalence of viral mRNA (26.32% bioMérieux and 34.21% Roche). Women that had suffered from lesion progression were mostly positive for HPV mRNA (94.4% bioMérieux and 58.82% Roche), while viral expression positivity was found to be lower in women that had remained with the same type of lesion (43.75% bioMérieux and 6.06% Roche). These results agreed with other authors that suggested E6/E7 mRNA expression as a prognostic factor for high-grade lesions and confirmed viral oncogene expression association with severity of cervical lesions [27–29].

We evaluated the concordance between bioMérieux and Roche with lesion type and evolution, applying two different coefficients, the relative observed agreement and Cohen’s kappa. The relative observed agreement was found to be from fair to nearly perfect for both extraction methods while Cohen’s kappa revealed a fair to moderate concordance. Agreement differences might be explained due to the low number of samples included in the study.

As reported by Ovestad et al., a test for screening population should have both high sensitivity and specificity values [30]. In our study, none of extraction methods showed high values for both parameters. While Roche method showed a higher PPV and specificity, bioMérieux revealed a higher sensitivity, NPV and mRNA positive rate. Moreover, regarding cost effectiveness, the cost of RNA extraction plus oncogenes detection was almost double for bioMérieux than Roche (8.5 and 4.9, respectively). Nowadays, there is not a gold standard test for oncogenes expression detection [31] and further studies are needed to elucidate and validate a standard protocol.

## Conclusions

We demonstrated the importance of validating a protocol for HPV E6 and E7 oncogene expression detection. Extraction methods based on magnetic particles provided better RNA yield than those based in columns. Both Nuclisens manual extraction kit (bioMérieux) and High Pure Viral RNA Kit (Roche) seemed to be adequate for E6/E7 mRNA detection. However, none of them revealed both high sensitivity and specificity values. Sensitivity was proven to be essential when detecting viral mRNA expression, as certain genotypes as well as episomal forms, might not produce a high number of transcripts to transform cells. Further studies are needed to obtain and validate a standard gold method for RNA expression detection, to be included as part of the routine cervical screening method.

## Abbreviations

ASCUS: Atypical squamous cells of undetermined significance
CIN: Cervical intraepithelial neoplasia
HPV: Human papillomavirus
HR-HPV: High-risk Human papillomavirus
HSIL: High-grade squamous intraepithelial lesion
LSIL: Low-grade squamous intraepithelial lesion
NPV: Negative predictive value
ORF: Open reading frame
PPV: Positive predictive value
